# Processing Language Partly Shares Neural Genetic Basis with Processing Tools and Body Parts

**DOI:** 10.1523/ENEURO.0138-24.2024

**Published:** 2024-08-01

**Authors:** Haojie Wen, Dahui Wang, Yanchao Bi

**Affiliations:** ^1^State Key Laboratory of Cognitive Neuroscience and Learning, Beijing Normal University, Beijing 100875, China; ^2^IDG/McGovern Institute for Brain Research, Beijing Normal University, Beijing 100875, China; ^3^Beijing Key Laboratory of Brain Imaging and Connectomics, Beijing Normal University, Beijing 100875, China; ^4^School of Systems Science, Beijing Normal University, Beijing 100875, China; ^5^Chinese Institute for Brain Research, Beijing 102206, China

**Keywords:** anterior temporal lobe, basal ganglia, body parts, genetic, language, superior temporal cortex, tools

## Abstract

Language is an evolutionarily salient faculty for humans that relies on a distributed brain network spanning across frontal, temporal, parietal, and subcortical regions. To understand whether the complex language network shares common or distinct genetic mechanisms, we examined the relationships between the genetic effects underlying the brain responses to language and a set of object domains that have been suggested to coevolve with language: tools, faces (indicating social), and body parts (indicating social and gesturing). Analyzing the twin datasets released by the Human Connectome Project that had functional magnetic resonance imaging data from human twin subjects (monozygotic and dizygotic) undergoing language and working memory tasks contrasting multiple object domains (198 females and 144 males for the language task; 192 females and 142 males for the working memory task), we identified a set of cortical regions in the frontal and temporal cortices and subcortical regions whose activity to language was significantly genetically influenced. The heterogeneity of the genetic effects among these language clusters was corroborated by significant differences of the human gene expression profiles (Allen Human Brain Atlas dataset). Among them, the bilateral basal ganglia (mainly dorsal caudate) exhibited a common genetic basis for language, tool, and body part processing, and the right superior temporal gyrus exhibited a common genetic basis for language and tool processing across multiple types of analyses. These results uncovered the heterogeneous genetic patterns of language neural processes, shedding light on the evolution of language and its shared origins with tools and bodily functions.

## Significance Statement

Human language entails a distributed brain network spanning across frontal, temporal, parietal, and subcortical regions. To elucidate the genetic basis underlying this complex language network, we adopted the Human Connectome Project functional magnetic resonance imaging twin data to examine the relationship between the genetic effects for the brain responses to language and to object domains that have been hypothesized to coevolve with language (tools, social, and body actions). The bilateral basal ganglia exhibited a common genetic basis for language, tool, and body part processing and the right superior temporal gyrus for language and tool processing. These results provide evidence for the heterogeneous genetic patterns of language neural processes and shed light on its potential origin with tools and bodily actions.

## Introduction

Language is an evolutionary signature of *Homo sapiens*, serving as a foundation for thinking and communication (Rilling et al., 2008; Friederici et al., 2017). Such ability is supported by a widely distributed brain network encompassing the left inferior and superior frontal cortex, the left inferior parietal lobe (IPL), the bilateral temporal lobes (Fedorenko et al., 2010; Barch et al., 2013), and subcortical regions, including the basal ganglia (Thibault et al., 2021; Murphy et al., 2022), which arguably have different functionalities underlying language processing, such as syntax, semantics, and speech.

Recent advances have been made in elucidating the genetic effects among the neural correlates underlying language processing. For task responses, Le Guen et al. (2018) showed that genetic factors accounted for substantial interindividual differences in language functional magnetic resonance imaging (fMRI) activation in regions including the superior temporal sulcus, the middle frontal gyrus (MFG), and the inferior frontal cortex. For the resting-state functional connectivity (rsFC) network, a correlation between gene expression and the language rsFC network was observed, along with a consensus set of genes that affected the overall connectivity-transcriptome correlation (Kong et al., 2020) and multiple genomic loci associated with language FCs (Mekki et al., 2022). These studies provide positive evidence that neural activity supporting language is at least partly genetically driven, yet whether the different regions within the complex large-scale language network, spanning across frontal, temporal, parietal, and subcortical regions, have common or different genetic mechanisms remains unknown. Answering this question helps us to characterize the genetic features of different language regions, providing clues about their functionality from a genetic perspective, and gain insights into the evolutionary mechanisms of language.

One approach to tackle the potential genetic patterns underlying the complex language network is by examining its relations with other cognitive functions that are hypothesized to coevolve with language in biological evolution. Language evolution has been hypothesized to be linked to several key cognitive abilities (though not in mutually exclusive manner; Dunbar, 1998, 2003; Fitch et al., 2010; Vaesen, 2012). One is complex tool use, with theories assuming that language evolved with complex tool use, sharing a key cognitive component of hierarchical processing of information sequences. Indeed, recent brain imaging studies have shown that tool use and comprehension of sentences requiring hierarchical processes (e.g., object relative clause) share neural activity (both activity strength and neural representations) in the basal ganglia and that learning effects transfer across these tasks (Higuchi et al., 2009; Vaesen, 2012; Brozzoli et al., 2019; Thibault et al., 2021). One is gesturing, a specific way of social communication that is considered a precursor or foundation for the evolution of language (Sterelny, 2012; Hobaiter and Byrne, 2014; Graham and Hobaiter, 2023). Finally, and not mutually exclusively, language evolution has been proposed to be motivated and intertwined with social interaction, with language relieving the pressure of social survival by replacing grooming behavior and sharing a cognitive mechanism with social cognition such as the theory of mind (Dunbar, 1998, 2003, 2017; Fitch et al., 2010; Oesch and Dunbar, 2017; Rajimehr et al., 2022). Investigating whether and how the genetic effects underlying neural activities in language processing are related to the genetic effects underlying these processes is one way to parse the potential genetic patterns underlying language neural networks.

Here, we examined the genetic effect patterns for language brain network questions by exploiting the Human Connectome Project (HCP) dataset, which had fMRI data from monozygotic (MZ) twins and dizygotic (DZ) twins performing a variety of tasks, which allows researchers to test the extent to which the neural activity to a certain task is driven by genetic or environmental variables. We first examined whether there is heterogeneity regarding the genetic effects underlying the neural activity in the widely distributed cortical and subcortical regions responding to language using the language comprehension task (listening to stories). The potential genetic commonality and heterogeneity across the related language regions were further validated by analyzing the human gene expression data (Allen Human Brain Atlas, AHBA). Critically, for those brain regions showing different genetic effects for the neural activities of language, we explored how they may associate with the genetic effects for processes that have been hypothesized to relate to language evolution: tool, gesture, and social processing. To this end, we leveraged the HCP working memory task that contrasted pictures of four salient object domains that have been shown in the literature to yield distinct cortical regions reliable across subjects and time (tools, body parts, faces and places; see Barch et al., 2013; Y. Wang et al., 2020; Cabral et al., 2022 for positive object domain results using this dataset). These domains arguably are the main information domains with evolutionary saliency for humans (Mahon and Caramazza, 2011; Bi et al., 2016). The processes have been hypothesized to be potentially important for language evolution (tool, gesturing, social) and are intrinsically embedded in these domain contrasts (tools, body parts, and faces).

## Materials and Methods

Combining fMRI activation experiments with a twin study design and gene expression data, we investigated the genetic effect patterns of the language brain network. Using the language task from the HCP, we first identified the patterns of how genetic effects influenced the neural activities in the cortical and subcortical regions during language processing. Then, we performed classification analyses to further validate the potential genetic commonality and heterogeneity across the related language regions using the human gene expression (AHBA) dataset. Finally, we tested whether the observed genetic effects were related to the neural responses to the related processing object domains (tools, body parts, and faces) for each language genetic cluster using the working memory task from the HCP. Multiple validation analyses were conducted, including voxel-level overlapping analysis, common path (CP) model analysis, analysis using an alternative atlas, and analysis controlling for handedness.

### Subjects

Functional images of subjects were obtained from the WU-Minn HCP carried out at Washington University in St. Louis (Van Essen et al., 2013; https://www.humanconnectome.org/study/hcp-young-adult). The dataset contained 486 twin subjects, including 149 genetically confirmed MZ twin pairs (87 of which were female pairs) and 94 genetically confirmed DZ twin pairs (60 of which were female pairs; 1 of which was a different-sex pair). For both the language task and the working memory task, we selected same-sex twin pairs who had corresponding functional images and did not exhibit excessive head motion (>2 mm maximum translation or 2° rotation), which resulted in 113 MZ pairs (65 of which were female, 29.3 ± 3.31 years old) and 58 DZ pairs (34 of which were female, 28.8 ± 3.46 years old) for the language task and 106 MZ pairs (60 of which were female, 29.3 ± 3.34 years old) and 61 DZ pairs (36 of which were female, 28.8 ± 3.51 years old) for the working memory task. Cross-task analyses were based on 101 MZ pairs (57 of which were female, 29.3 ± 3.34 years old) and 51 DZ pairs (30 of which were female, 28.8 ± 3.48 years old) who participated in both tasks. The HCP was reviewed and approved by the Institutional Ethics Committee of Washington University in St. Louis, Missouri. All participants signed written informed consent documents.

### Experimental design

#### Language task

During the language task, fMRI data were acquired when the story blocks or math blocks were presented to subjects over two runs. Each run contained four language blocks (average of ∼30 s) and multiple math blocks (match the length of the story task blocks). In the story task, participants were presented with auditory stories (5–9 sentences) adapted from Aesop's fables, followed by a two-alternative forced–choice question related to the story. An example given in the original paper in which the task was described is “after a story about an eagle that saves a man who had done him a favor, participants were asked, ‘That was about revenge or reciprocity?’” (Binder et al., 2011). In the math task, participants were presented with arithmetic operations (e.g., “fourteen plus twelve”), followed by “equals” and then two choices (e.g., “twenty-nine or twenty-six”).

#### Working memory task

During the working memory task, fMRI data were acquired when subjects viewed pictures from the four object domains (i.e., tools, faces, body parts, and places; Barch et al., 2013) over two runs. Each run contained eight task blocks (25 s each, two for each object domain) and four fixation blocks (15 s each). Each block contained 10 trials (2.5 s each) presenting pictures from a single-object domain, and each picture was presented for 2 s, followed by a 500 ms intertrial interval. A 2.5 s cue indicating the task type (and target for zero-back) was shown at the start of the block. Participants were instructed to judge whether each image shown matched the target image (i.e., zero-back task) or whether each image shown matched the one shown two images back (i.e., two-back task) and to press the button under the index finger for “match” and the button under the middle finger for “no match.” Within each run, the zero-back and two-back tasks were presented once for each object domain.

### Image acquisition

Images were collected using a 3 T Siemens Skyra magnetic resonance scanner with a 32-channel head coil (Van Essen et al., 2013). Functional images were collected using a gradient-echo echo–planar imaging sequence with the following parameters: repetition time (TR), 720 ms; echo time (TE), 33.1 ms; flip angle (FA), 52°; bandwidth, 2,290 Hz/pixel; field of view (FOV), 208 × 180 mm^2^; matrix, 104 × 90; voxel size, 2 × 2 × 2 mm^3^; multiband (MB) factor, 8; slices, 72; and total scan time of 405 frames (5 min and 1 s) for the working memory task and 316 frames (3 min and 57 s) for the language task. For both tasks, two runs were collected with both phase encoding directions (i.e., left-to-right and right-to-left). High-resolution T1–weighted images were also acquired for every participant using a magnetized rapid gradient-echo imaging (MPRAGE) sequence with TR, 2,400 ms; TE, 2.14 ms; reversal time, 1,000 ms; FA, 8°; FOV, 224 × 224 mm^2^; voxel size, 0.7 mm isotropic; and total scan time, 7 min and 40 s.

### Data preprocessing

We used the minimally preprocessed images whose pipeline included gradient unwarping, motion correction, fieldmap-based EPI distortion correction, brain-boundary–based registration of EPI to structural T1-weighted scan, nonlinear (FNIRT) registration into MNI152 space, and grand-mean intensity normalization (see Glasser et al., 2013 for detailed preprocessing procedures). These images were further spatially smoothed with a 4 mm FWHM Gaussian kernel using the Statistical Parametric Mapping software (SPM12; http://www.fil.ion.ucl.ac.uk/spm/software/spm12/). Twin pairs that did not have corresponding functional images or exhibited excessive head motion (>2 mm maximum translation or 2° rotation) were excluded from further analysis.

### Data analysis

For each individual subject, the preprocessed task functional images were entered into a general linear model (GLM). For each run of the language task, the GLM included two regressors of interest corresponding to the story and math tasks, each convolved with the canonical hemodynamic response function (HRF). The GLM also included 12 head motion parameters as regressors of no interest. The high-pass filter was set at 128 s. After model estimation, the whole-brain *t* maps of language versus baseline were calculated across runs for each subject (Glasser et al., 2016; Rajimehr et al., 2022).

For each run of the working memory task, we construct the object domain contrasts without differentiating the task conditions (i.e., zero-back and two-back), in accordance with the HCP manual (https://www.humanconnectome.org/storage/app/media/documentation/s1200/HCP_S1200_Release_Reference_Manual.pdf). The GLM included four regressors of interest corresponding to the four object domains, each convolved with the canonical HRF. The GLM also included 12 head motion parameters as regressors of no interest. The high-pass filter was set at 128 s. To mitigate the influence of working memory and focus on our interests—tools, body parts, and faces—whole-brain *t* maps of those three object domains versus the place were calculated across runs for each subject.

### Definition of language regions

The language functional activation map was defined using the language versus baseline contrast under a threshold of voxel-level *p* < 0.001, one-tailed, cluster-level family–wise error (FWE)-corrected *p* < 0.05. We utilized the connectivity-based parcellation atlas (246 regions in total; Fan et al., 2016) to divide the functional map into 40 distinct language regions. Specifically, regions of the connectivity-based parcellation containing >100 activated voxels were defined as language regions. As the basal ganglia were involved in language processing (Thibault et al., 2021; Murphy et al., 2022) and given the limited signal-to-noise ratio and relative small size of its subregions, we applied a more lenient threshold (>40 activated voxels) to select the regions of interest (ROIs) of the basal ganglia. See Table 1 for the full list of the identified language regions.

**Table 1.** Language genetic effect results for all the language regions

Region number	Mask label	MZ twin		DZ twin		rMZ-rDZ	2*rDZ-rMZ	Initial model	Best model	ΔAIC	*p*	Cluster
		*r*	*p*	*r*	*p*							
1	SFG_L_7_1	0.28	0.001	0.11	0.194	0.17	−0.06	ADE	DE	7.63	0.002	5
2	MFG_L_7_2	0.25	0.004	0.41	0.001	−0.16	–	ACE	CE	13.86	***	–
3	IFG_L_6_1	0.18	0.029	0.21	0.051	−0.03	–	ACE	CE	4.03	0.014	–
4	IFG_L_6_2	0.34	0.000	0.15	0.134	0.19	−0.04	ADE	DE	12.90	***	5
5	IFG_L_6_3	0.23	0.007	0.06	0.325	0.17	−0.11	ADE	DE	4.43	0.011	5
6	IFG_L_6_4	0.27	0.002	0.10	0.227	0.17	−0.07	ADE	DE	7.19	0.002	5
7	IFG_L_6_5	0.26	0.002	0.18	0.084	0.08	0.10	ACE	*E*	–	–	–
8	ORG_L_6_2	0.26	0.003	0.23	0.040	0.03	0.20	ACE	CE	8.59	0.001	–
9	ORG_L_6_6	0.26	0.003	0.26	0.021	0.00	–	ACE	CE	9.60	***	–
10	PrG_L_6_1	0.09	0.163	0.04	0.370	0.05	−0.01	ADE	*E*	–	–	–
11	PrG_L_6_3	0.16	0.047	0.30	0.010	−0.14	–	ACE	CE	5.58	0.006	–
12	STG_L_6_1	0.21	0.013	0.17	0.099	0.04	0.13	ACE	CE	5.15	0.008	–
13	STG_L_6_2	0.30	0.001	−0.03	0.586	0.33	−0.36	ADE	DE	7.82	0.002	4
14	STG_R_6_2	0.26	0.002	−0.13	0.833	0.39	−0.52	ADE	DE	4.68	0.01	4
15	STG_L_6_3	0.23	0.008	−0.09	0.752	0.32	−0.41	ADE	DE	3.14	0.023	4
16	STG_R_6_3	0.32	0.000	−0.09	0.747	0.41	−0.50	ADE	DE	9.39	***	4
17	STG_L_6_4	0.27	0.002	0.06	0.335	0.21	−0.15	ADE	DE	6.74	0.003	2
18	STG_R_6_4	0.30	0.001	−0.13	0.842	0.43	−0.56	ADE	DE	7.04	0.003	4
19	STG_L_6_5	0.30	0.001	0.10	0.229	0.20	−0.10	ADE	AE	8.64	0.001	1
20	STG_R_6_5	0.33	0.000	0.02	0.444	0.31	−0.29	ADE	DE	11.10	***	3
21	STG_L_6_6	0.32	0.000	0.14	0.145	0.18	−0.04	ADE	AE	11.14	***	2
22	STG_R_6_6	0.41	0.000	0.00	0.513	0.41	−0.41	ADE	DE	17.21	***	3
23	MTG_L_4_2	0.37	0.000	0.09	0.252	0.28	−0.19	ADE	DE	15.37	***	1
24	MTG_R_4_2	0.37	0.000	0.09	0.243	0.28	−0.19	ADE	DE	15.82	***	1
25	MTG_L_4_4	0.41	0.000	0.15	0.130	0.26	−0.11	ADE	DE	19.96	***	1
26	MTG_R_4_4	0.38	0.000	0.01	0.475	0.37	−0.36	ADE	DE	14.64	***	3
27	ITG_L_7_3	0.36	0.000	0.04	0.383	0.32	−0.28	ADE	DE	13.22	***	1
28	FuG_L_3_1	0.06	0.247	0.33	0.005	−0.27	–	ACE	AE	0.15	0.14	–
29	pSTS_L_2_1	0.38	0.000	−0.03	0.588	0.41	−0.44	ADE	DE	14.72	***	2
30	pSTS_R_2_1	0.31	0.000	−0.10	0.772	0.41	−0.51	ADE	DE	7.49	0.002	3
31	pSTS_L_2_2	0.30	0.001	−0.13	0.832	0.43	−0.56	ADE	DE	6.83	0.003	2
32	IPL_L_6_5	0.26	0.002	0.12	0.180	0.14	−0.02	ADE	DE	6.95	0.003	5
33	IPL_L_6_6	0.15	0.058	−0.03	0.600	0.18	−0.21	ADE	DE	0.11	0.15	–
34	PoG_L_4_2	0.14	0.062	0.05	0.343	0.09	−0.04	ADE	DE	0.35	0.13	–
35	PoG_R_4_2	0.08	0.201	0.00	0.508	0.08	−0.08	ADE	*E*	–	–	–
36	Hipp_L_2_1	0.00	0.486	0.09	0.243	−0.09	–	ACE	*E*	–	–	–
37	BG_L_6_1	0.04	0.341	−0.04	0.611	0.08	−0.12	ADE	*E*	–	–	–
38	BG_L_6_3	−0.08	0.806	−0.13	0.832	0.05	–	ACE	*E*	–	–	–
39	BG_L_6_5	0.28	0.001	0.10	0.215	0.18	−0.08	ADE	AE	8.10	0.001	6
40	BG_R_6_5	0.24	0.005	−0.14	0.847	0.38	−0.52	ADE	DE	3.79	0.016	6

Note: *** denotes *p* < 0.001.

### Genetic modeling of language regions

To examine the genetic influence on each language region, we utilized structural equation models implemented with the umx package (Bates et al., 2019). The estimated components of the model included additive genetic factor (*A*); dominance genetic factor (*D*); common environmental factor (*C*); and unique environmental factor, including measurement errors (*E*). As factors *C* and *D* are confounded in the classical twin study, they cannot be accurately estimated simultaneously (Rijsdijk, 2002). Therefore, the most appropriate initial model (ACE or ADE) for each language region was first determined by comparing the intraclass correlation (ICC) in the MZ and DZ group. If the DZ correlations were less than half the MZ correlations, factor *D* was indicated; otherwise, factor *C* was chosen.

For each language region, activation indexes (i.e., *t* values) were fitted to the initial model and its submodels, which were subsequently compared with each other. For instance, when using the ACE model, we compared it with the AE, CE, and *E* models. Model-fit metrics (the Akaike information criterion, AIC) were computed for each model, and the best model was chosen based on the lowest AIC value. For the best model that included a genetic factor, a higher ICC between MZ pairs than between DZ pairs was also required. The goodness-of-fit *χ*^2^ between the best model and control models was computed, and regions exhibiting significant effects (*p* < 0.05) of the genetic factor and the common environmental factor were defined as the genetically influenced language regions and environmentally influenced language regions, respectively.

To streamline our analysis and avoid redundancy, we grouped the genetically influenced language regions into language clusters using the CP model. The CP model allowed us to test whether genes and the environment worked through one or multiple common latent factors (Bates et al., 2019). Here, we applied the CP model to all genetically influenced language regions, with the optimal number of common factors determined by model-fit metrics and goodness-of-fit *χ*^2^. Based on the observation that most genetically influenced language regions were associated with dominance genetic factors (Table 1), we chose the ADE model as the CP model. The different common factors represented the different shared components extracted from the activity of all genetically influenced language regions. For each genetically influenced language region, we used its loading values on each common factor as a vector to represent itself. Using the *k*-means clustering method, all the genetic regions were split into multiple language genetic clusters. The optimal number of clusters was established using the “gap_stat” method.

### Gene expression of language cluster analyses

To validate the commonality and differences underlying the genetic effects in the language genetic clusters, we analyzed postmortem human brain genome expression data obtained from the AHBA dataset (http://www.brain-map.org, Hawrylycz et al., 2012; Sunkin et al., 2012). To date, six adult donors with no history of neuropsychiatric or neurological conditions are available in the database. As our focus was on both hemispheres, our analyses were primarily based on donors who had data from both hemispheres (*N* = 2). Validation analyses were also performed on data from the six donors when necessary. The microarray data were preprocessed using a state-of-the-art analysis pipeline (Arnatkevic̆iu¯tė et al., 2019) that included data filtering, probe selection, sample assignment, and gene filtering. Through preprocessing, the gene expression data were normalized across probes and donors and within a given region, resulting in a single expression measure of each region for each gene. The ROIs were defined using the connectivity-based parcellation atlas and normalized to individual donors using normalization offered by Advanced Normalization Tools (Avants et al., 2009, https://stnava.github.io/ANTs). Regions with no gene expression data were removed from further analysis. Additionally, we ensured the data's high differential stability (top 5%, reflecting interindividual consistency) as per Kong et al. (2020) to maintain data quality. This led to the creation of a region × gene expression data matrix that can be utilized for subsequent analysis.

The linear support vector machine (SVM; LIBSVM, http://www.csie.ntu.edu.tw/∼cjlin/libsvm; Chang and Lin, 2011) with standard parameters was used in a leave-region-out cross–validation scheme for the gene expression pattern classification analyses. Two types of decoding analyses were performed: one is to decode between genetically influenced language regions and nonlanguage regions to characterize the potential commonalities of different language clusters; another was to decode among different language genetic clusters to assess differences among language genetic clusters.

For the first type of decoding analysis, we randomly sampled 23 nonlanguage regions with the same number as the genetically influenced language regions (two regions from Cluster 5 were excluded due to a lack of genetic expression data). Each region had 501 features. In 46 iterations, we trained a classifier using 45 regions as training data and tested it on the remaining region. Classification accuracy was computed and averaged across 46 iterations. To avoid regional selection errors and enable statistical testing, this operation was repeated 10,000 times. Genes with high weights (top 1%) were deemed important in classification.

For the second type of decoding analysis, a classifier was trained to classify six different clusters. In each of the 23 iterations, 22 regions served as training data, and 1 region was reserved as testing data. Classification accuracy was measured by averaging the results across all the 23 iterations. To establish the statistical significance of the classification accuracy, we conducted a permutation analysis. For this analysis, the cluster labels were randomly assigned, and a new classification accuracy value was calculated based on the generated data. This randomized process was repeated 10,000 times, resulting in a distribution which was used to establish the statistical confidence of the actual accuracy obtained from the original data. To identify genes unique to each cluster, we used all sample data points and employed two-sample independent *t* tests to directly compare and determine the gene with the greatest difference between specific clusters and other clusters.

### Object domain genetic influence analyses

To assess in what way the observed genetic effects are related to the neural responses to related processing of object domains, we applied genetic model analyses to the activation (i.e., *t* values) of multiple object domains (tools, body parts, and faces) for each cluster. We performed permutation analysis to examine the significance of the difference between the best model (e.g., DE model) and the control model (e.g., *E* model). Null distributions of the model difference were generated by permuting the MZ/DZ labels and shuffling the twin pairs. This permutation was repeated 10,000 times, and the best genetic model, as well as the *E* model, was estimated. The likelihood differences (ΔAIC) between the best genetic model and the *E* model were computed to form the distribution. To ascertain whether the observed genetic effects were simply due to the presence of domain-responsive voxels within the language clusters, we defined the object domain map using each domain versus place contrast under a threshold of voxel-level *p* < 0.001, one-tailed, cluster-level FWE–corrected *p* < 0.05. Subsequently, we excluded these voxels from the language clusters and recalculated the genetic effects.

### Validation analyses

Multiple lines of analyses were conducted to further examine the genetic effect related to the neural response to object domains (tools, body parts, and faces) for each language cluster.

#### Voxel-level overlapping analysis

The first validation analysis entailed testing whether each language cluster exhibited a genetic effect on a specific object domain when the analyses were restricted to the “language genetic voxels.” “Language genetic voxels” were defined as follows: for each voxel in the language cluster, genetic models were constructed and compared using a procedure similar to the Genetic modeling of language regions section; voxels, for which the best-fitting model included the genetic factor (*A* or *D*) and showed significant improvement over control models (*p* < 0.05), were defined as “language genetic voxels.”

#### CP model analysis

The second validation analysis was performed to investigate whether common factors influenced by genetics existed between language and specific object domains. CP models were applied to subjects who completed both the language task and the working memory task. For each language genetic cluster, object domains that exhibited genetic influence were combined with language data to fit a CP model. Given most language genetic clusters reported in the main results have exhibited the effect of the dominance genetic factor, ADE models were primarily considered in the CP model. The number of common factors was one, as additional factors did not significantly improve the model fit. A shared genetic influence between language and certain object domains was demonstrated by the presence of a common factor with genetic influence (*A* or *D*).

#### Anatomically defined region validation analysis

The third validation analysis was conducted to examine the robustness of the two clusters we identified across different parcellations. We performed a validation analysis using the HCPex template (Huang et al., 2022). The HCPex template is a modified and extended version of the original atlas (HCP-MMP v1.0; Glasser et al., 2016) in which 66 subcortical areas (33 in each hemisphere) including the basal ganglia have been added. We selected the regions for two language clusters of interest according to the anatomical location and reestimated the genetic effects of language and object domain processing. More specifically, the bilateral dorsal caudate (dCa) cluster corresponded to the combination of Caudate_L and Cauduate_R regions. The right STS cluster corresponded to the combination of Area_STGa_R, Area_STSd_anterior_R, Area_STSd_posterior_R, Area_STSv_anterior_R, and Area_STSv_posterior_R.

#### Validation analysis regressing out handedness

The fourth validation analysis was performed to eliminate the influence of handedness. To include as many subjects as possible, we did not distinguish between different handedness groups in the main analysis (Schmitt et al., 2014; Le Guen et al., 2018). To ensure the results were not affected by handedness, we estimated the genetic models with handedness information (provided by the HCP) as a control variable and computed the goodness-of-fit *χ*^2^ to assess the statistical significance.

### Data and code availability

The fMRI dataset and AHBA dataset are available at https://www.humanconnectome.org and https://human.brain-map.org, respectively. Due to HCP privacy policies, the preprocessed images (with their IDs) can only be shared upon request with qualified investigators who agree to the Restricted Data Use Terms of the dataset. The codes supporting the findings of this study are available from the corresponding authors upon reasonable request.

## Results

### Genetic influence found in multiple cortical and subcortical language regions

**Figure 1.**
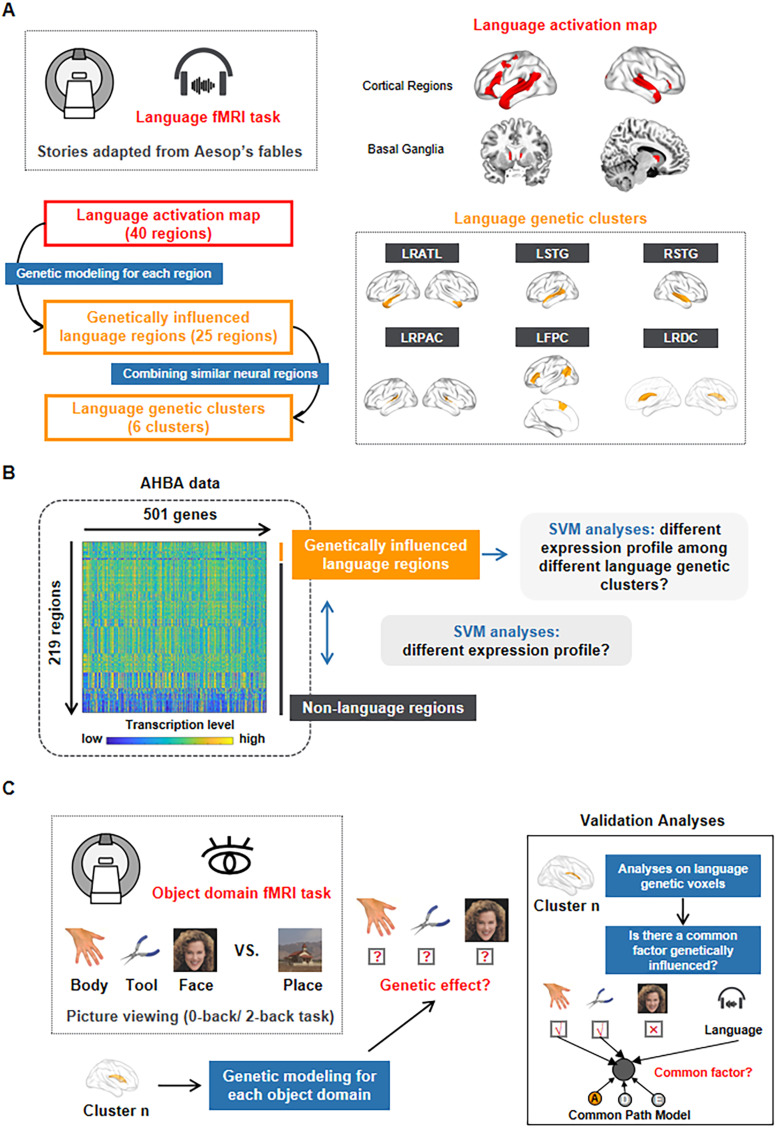
A flow chart of the neural genetic investigation analysis for language. ***A***, The definition of language activation regions and language genetic clusters. The top-right panel displays the language activation map, which was derived from the HCP language fMRI task. In this task, subjects listened to stories adapted from Aesop's fables and answered questions related to them (as shown in the top-left panel). The language map was further subdivided into 40 regions using the Brainnetome Atlas (Fan et al., 2016). For each region, genetic modeling was performed, and the regions with a genetic factor (*A* or *D*) were identified as genetically influenced language regions. Then, 25 genetically influenced language regions were merged based on neural activation, resulting in six language genetic clusters (as shown in the bottom-right panel). Slice views and projected brain images were prepared in MRIcron (https://www.nitrc.org/projects/mricron) and BrainNet Viewer (Xia et al., 2013), respectively. ***B***, Gene expression analyses of language clusters. The left panel displays the region × gene expression matrix, which was generated through multiple preprocessing steps and used for subsequent analyses. Two types of decoding were conducted: decoding between genetically influenced language regions and nonlanguage regions to test whether the language genetic clusters exhibited systematically different genetic profiles compared with the rest of the brain and decoding among different language genetic clusters to examine differences among language genetic clusters. The genes that contributed to these two classifications were identified accordingly. ***C***, The investigation of genetic factors for multiple object domains. The genetic impact of each language cluster on various object domains, including tools, body parts, and faces, was evaluated using the HCP working memory task. In this task, subjects viewed pictures from four object domains (i.e., tools, faces, body parts, and places; Barch et al., 2013) over two runs (shown in the left panel). To avoid the influence of working memory and focus on our interests, tools, body parts, and faces, whole-brain contrast images of these three object domains versus places were calculated and measured at the individual level after model estimation. Multiple lines of analyses (shown in the right panel) were conducted to further test the genetic relationship between language and other object domains for each language genetic cluster. The first validation analysis tested whether each language cluster exhibited a genetic effect on specific object domains when the analyses were restricted to the “language genetic voxels.” The second validation analysis investigated whether there were common factors influenced by genetics between language and specific object domains with the existing genetic influence through the CP model.

We utilized the language task dataset from the HCP (Van Essen et al., 2013) to define the activation regions for language and to estimate the genetic influence on such activation. The language task involved 342 subjects from 171 twin pairs, including 113 pairs of genetically confirmed MZ twins and 58 pairs of genetically confirmed DZ twins. In the language condition, subjects listened to stories (5–9 sentences) adapted from Aesop's fables and answered a two-alternative forced–choice question related to the story. The activation map for language was established by comparing significant differences between language and baseline conditions (as shown in Fig. 1*A*, voxel-level *p* < 0.001, cluster-corrected using FWE). The map included widely distributed cortical clusters in the left frontal lobe including the inferior frontal gyrus (IFG); MFG; superior frontal gyrus (SFG); orbital gyrus (OrG) and precentral gyrus (PrG); bilateral temporal lobes including the anterior temporal cortex, middle temporal cortex, and posterior temporal cortex; and left parietal lobes including the IPL and postcentral gyrus (PoG), corresponding well to the language network identified using different datasets (Fedorenko et al., 2010), primary auditory cortex, and subcortical regions (the hippocampus and basal ganglia). We partitioned this functionally identified language mask into 40 distinct (language) regions using the Brainnetome atlas (Fan et al., 2016; Fig. 1*A*; see details in Table 1).

**Figure 2.**
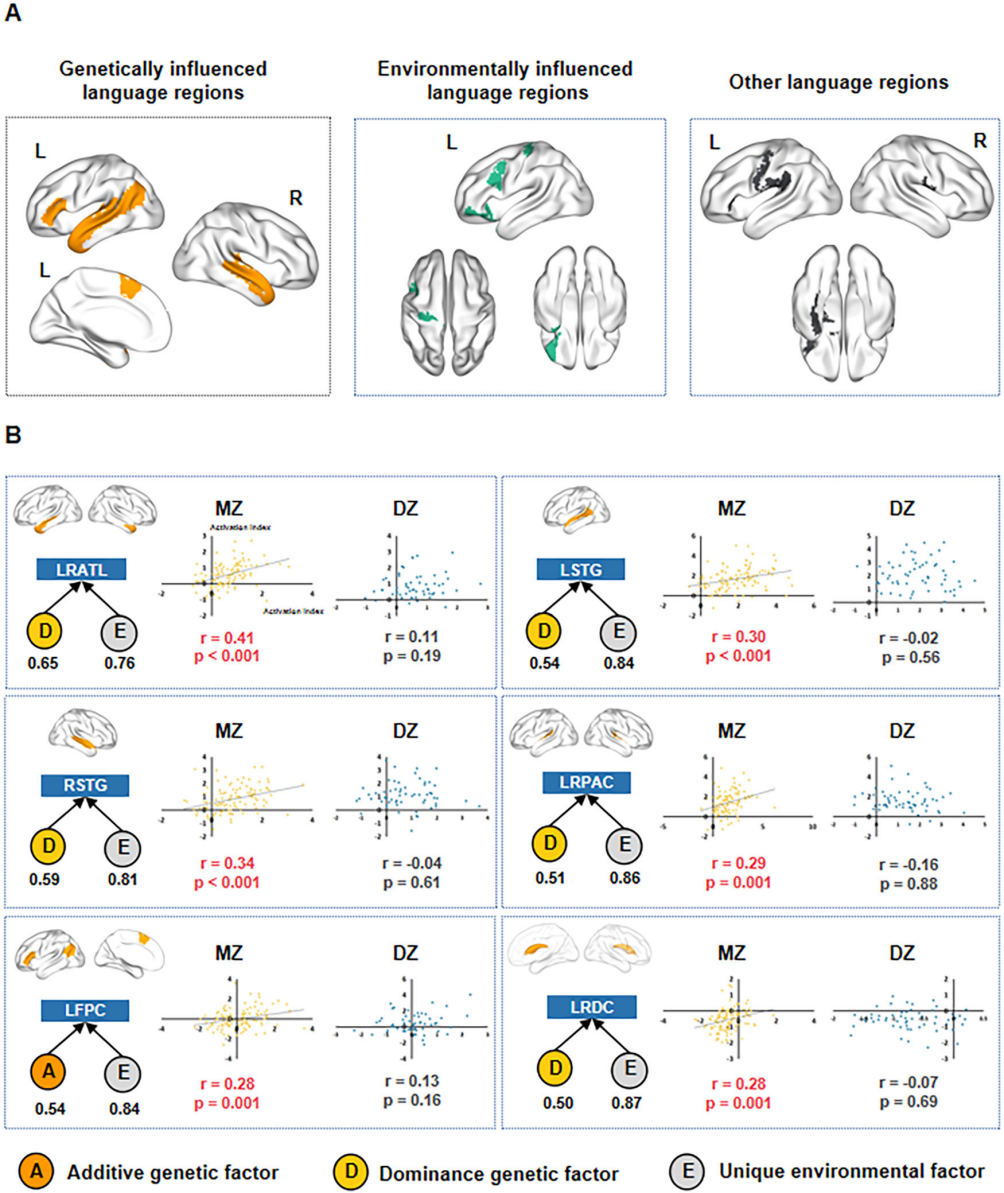
Genetic influence found in multiple cortical and subcortical language regions. ***A***, Multiple types of language regions. After genetic modeling, multiple types of language regions were defined. The left panel shows the genetically influenced language regions, which were identified by the presence of a significant genetic factor (*A* or *D*). Out of 25 language regions, 22 regions were best modeled by model DE, and 3 regions were best modeled by model AE (*p* < 0.05, compared with the control model *E*). The middle panel shows the environmentally influenced language regions, which were identified by having a significant common environmental factor (*C*) in the model CE. Six language regions showed significant common environment effects (*p* < 0.05, compared with the control model ***E***). The right panel shows the other language regions, which did not have any significant genetic or environmental factors in the models. *A*, *D*, and *E* denote the additive genetic factor, dominance genetic factor, and unique environment factor, respectively. See language genetic influence result without a language mask in Extended Data Figure 2-1. ***B***, Genetic influence found in the six language clusters. For each language cluster, the left top and bottom panels show the ROI and best model for each language cluster, respectively. *A*, *D*, and *E* denote the additive genetic factor, dominance genetic factor, and unique environment factor, respectively, and their values are indicated below each factor. The scatterplots show the ICC across the subject pairs in the MZ and DZ groups. Each dot represents a twin pair in each group.

For each language region, we estimated whether the activity strength in response to language stimuli was driven by genetic variables. We applied genetic models—ACE, ADE, and their submodels—to the language data acquired from the twins and tested whether the best-fit model contained a genetic factor (i.e., additive genetic factor, *A*, or dominance genetic factor, *D*). Out of the 40 language regions, 25 were found to be influenced by genetic factors, with 22 regions being best modeled as DE and 3 as AE: cortical regions, including the left SFG, left IFG, left IPL, bilateral superior temporal gyrus (STG), bilateral middle temporal gyrus (MTG), left inferior temporal gyrus (ITG), and bilateral posterior superior temporal sulcus (pSTS), as well as the bilateral subcortical region basal ganglia (dCa; Fig. 2*A*). Not only did these data reveal AE or DE as the best models (based on AIC), but they also showed a significant advantage over the control *E* model (*p*s < 0.05). We also conducted permutation analyses to test the significance of all genetic regions, and all the best models showed a significant advantage over the control *E* model (all *p* < 0.05) under false discovery rate (FDR) multiple-comparison correction. Additionally, six language regions were found to be significantly influenced by common environmental factors (i.e., *C*, model CE was the best model, and *p*s < 0.05 when compared with the control model *E*), localized in the left MFG, left IFG, left OrG, left PrG, and left STG (Fig. 2*A*). For the other nine language regions (one in the left IFG, one in the left PrG, one in the left FuG, one in the left IPL, two in the left PoG, one in the left hippocampus, and two in the bilateral basal ganglia), no significant effects of either genetic or common environmental factors were observed.

10.1523/ENEURO.0138-24.2024.f2-1 Figure 2-1 **Language genetic influence result without a language mask** **A. Multiple types of whole-brain regions.** After genetic modeling without restricting the language activation map, multiple types of regions were identified. **B. The clustering result of the genetic regions without a language activation mask.** Download figure 2-1, DOCX file.

To streamline our analysis and avoid redundancy, we utilized the CP model and clustering methods to group these 25 language regions with genetic influence based on their language-related activities. Specifically, 11 components were derived from the CP model across all the genetically influenced language regions, and the loadings on each component for each genetically influenced language region were used to represent its corresponding region. The *k*-means clustering was conducted on all the genetically influenced language regions, with the optimal cluster number determined using the “gap_stat” method. Six clusters were obtained: the bilateral anterior temporal lobe (Cluster 1, LRATL), left STG (Cluster 2, LSTG), right STG (Cluster 3, RSTG), bilateral primary auditory cortex (Cluster 4, LRPAC), left IFG/SFG-IPL (Cluster 5, LFPC), and bilateral dCa (Cluster 6, LRDC; Fig. 1*A*). Confirmatively, the best models for all six clusters contained either an *A* or a *D* factor, as shown in Figure 2*B* (model AE or model DE, with all FDR-corrected *p* < 0.01; ΔAIC > 5; compared with model *E*). The genetic effects were also evidenced by the stronger ICCs between MZ twin pairs than between DZ twin pairs for each cluster (Fisher's *Z* test, mean *z* = 2.03; range, 0.97–2.73), as visualized in the scatterplots in Figure 2*B*. Note that while LRDC and LRPAC are not traditional language clusters (e.g., Fedorenko et al., 2010), we termed all of these clusters “language genetic clusters” to be inclusive, as they were similarly obtained in the current language mask identification.

We also showed the whole-brain genetic map without the constraint of a language activation mask (Extended Fig. 2-1*A*). Overall convergent patterns were obtained for genetically influenced regions, common environmentally related regions, and other regions influenced by unique environments (including measuring error). We clustered the genetically influenced regions without a language activation mask using a similar method (11 components, six clusters), resulting in clusters a–f shown in Extended Data Figure 2-1*B*. While some language genetic clusters were merged into one cluster or split into different clusters along with other nonlanguage activation regions in this clustering, the overall pattern converged well with those within the language mask. We focused on the language genetic clusters in the language activation mask below.

### Gene expression patterns across language genetic clusters: commonalities and differences

To validate the commonality and differences underlying the genetic effects in the language genetic clusters, we explored their gene expression patterns. To this end, we used postmortem genome expression data from the AHBA dataset (https://human.brain-map.org/), where >20,000 genes taken from 3,702 spatially distinct tissue samples from six human brains were measured. Left hemisphere data were available for all six donors, whereas two-hemisphere data were available for two donors. We preprocessed the data using a state-of-the-art analysis pipeline (Arnatkevic̆iu¯tė et al., 2019) that included data filtering, probe selection, sample assignment, and gene filtering. Through preprocessing, the gene expression data were normalized across probes and donors and within a given region, resulting in a single expression measure of each region for each gene. We ensured the high differential stability of the data (501 genes, top 5%, reflecting interindividual consistency) as per Kong et al. (2020) to maintain data quality.

**Figure 3.**
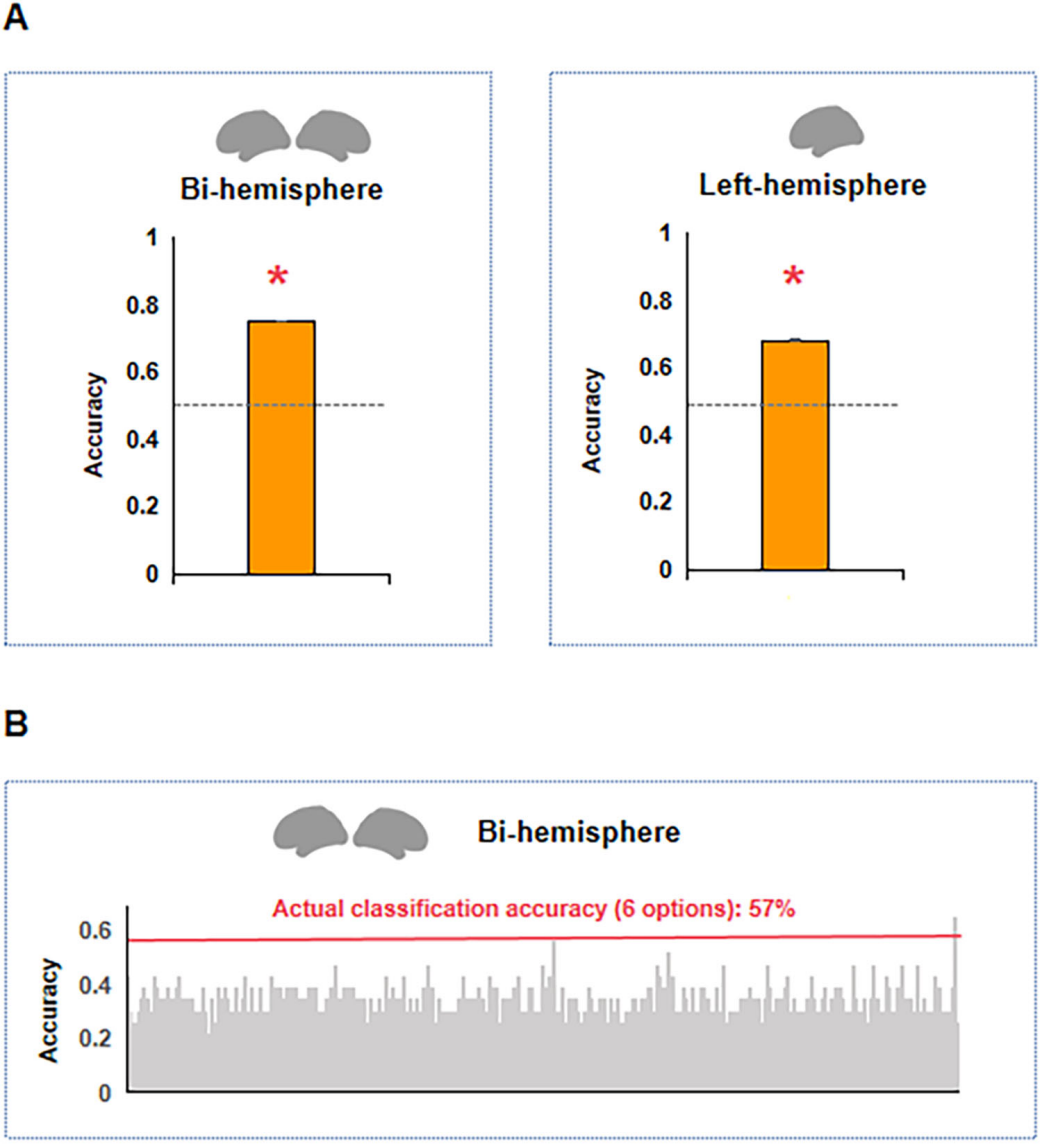
Gene expression patterns across language genetic clusters. ***A***, Common genetic expression profiles revealed by decoding between genetically influenced language regions and other nonlanguage regions. Bar graphs represent the average accuracy of the classification between genetically influenced language regions and nonlanguage regions over 10,000 iterations. Error bars indicate the standard error. The left panel displays the classification result based on the two hemispheres. The right panel shows the classification result based on the left hemisphere. The gray line represents the chance level. See functional labels based on Neurosynth for genes contributing to commonality of language clusters in Extended Data Table 3-1. ***B***, Different genetic expression profiles revealed by decoding between different language genetic clusters. The red line represents the actual accuracy of the classification among regions of different language genetic clusters. The distribution represents the accuracy of classification after disrupting labels and repeating the process 10,000 times. See functional labels based on Neurosynth for genes contributing to differences of different language clusters in Extended Data Table 3-2.

We first tested whether the language genetic clusters exhibited systematically different genetic profiles compared with the rest of the brain to characterize the potential commonalities of different language clusters. We used a SVM classification approach, examining whether the genetically influenced language regions (two donors, 23 regions, 2 regions from Cluster 5 were excluded due to a lack of gene expression data) versus those regions that were not activated by language (196 regions) could be successfully classified based on gene expression data in a leave-one-region-out fashion. A mean accuracy of 0.75 was obtained, which was significantly higher than chance (0.50, *p* < 0.001). That is, there was a significant difference between the gene expression profiles between genetically influenced language regions and the rest of the brain. Extended Data Table 3-1 shows genes with the highest 1% classifier weights, i.e., those providing the strongest contribution to the classification between genetically influenced language regions and nonlanguage regions. We also present the functions of these genes referenced by the Neurosynth-Gene function, which were based on a meta-analysis of group-average volume–based data (Yarkoni et al., 2011). We further performed the same analyses using data from the left hemisphere of six donors, and the results remained consistent (Fig. 3*A*; classification accuracy, 0.68, significantly higher than the 0.50 chance level; *p* < 0.001).

10.1523/ENEURO.0138-24.2024.t3-1 Table 3-1 **Functional labels based on Neurosynth for genes contributing to commonality of language clusters** Note: For each gene, the functional terms from Neurosynth represent the terms with the most similar meta-analysis whole-brain activation map to the gene’s whole-brain map. The correlation values indicate the correlation of the gene’s whole-brain expression with the term’s meta-analysis result. Download Table 3-1, DOC file.

10.1523/ENEURO.0138-24.2024.t3-2 Table 3-2 **Functional labels based on Neurosynth for genes contributing to differences of different language clusters** Note: For each gene, the functional terms from Neurosynth represent the terms with the most similar meta-analysis whole-brain activation map to the gene’s whole-brain map. The correlation values indicate the correlation of the gene’s whole-brain expression with the term’s meta-analysis result. Download Table 3-2, DOC file.

We next examined whether distinct genetic expression patterns existed among the six language genetic clusters. SVM was used for one in six classifications. In each of the 23 iterations, 22 regions served as training data, and 1 region was reserved as testing data. Classification accuracy was measured by averaging the results across all the 23 iterations. An accuracy of 0.57 was obtained, which was significantly higher than chance (Fig. 3*B*; chance level determined by a mean accuracy of 10,000 permutations, 0.13; *p* < 0.001). That is, there were systematically distinct gene expression patterns associated with different language genetic clusters. We repeated this analysis using data from the left hemisphere of six donors and observed consistent results (mean accuracy, 0.63; chance level determined by a mean accuracy of permutation of 10,000 times, 0.18; *p* < 0.001). To identify genes that were specifically associated with each language genetic cluster, we used all samples (whole brain, two donors) and compared the gene expression between each language genetic cluster and the remaining five using a *t* test. Extended Data Table 3-2 shows the genes with significantly higher expression levels in each cluster (top five genes for each cluster, *t*s with the remaining clusters >3.7; *p* < 0.001; see their functions provided by the Neurosynth-Gene function map in Extended Data Table 3-2).

### Differential genetic influence on responses to tools and body parts in different language clusters

Having established the heterogeneity among the language genetic clusters, we tested whether their genetic effects have different origins by examining their association patterns with the genetic effects underlying processes that have been hypothesized to relate to language evolution: tools, gestures, and social processes. The HCP working memory dataset was also designed to contrast salient object domains, which were highly related to these processes—pictures of tools, body parts, and faces (Fig. 1*C*). For each language genetic cluster, we thus tested whether they also exhibit genetic effects in terms of neural activity in the processing of tools, body parts, or faces. The current analysis included 334 subjects (167 twin pairs), of which 106 pairs were genetically confirmed MZ twins and 61 pairs DZ twins (see Materials and Methods for data inclusion criteria). Activities (*t* values) for the tools, body parts, and faces were obtained by contrasting the values of each object domain versus the place condition as a control, without differentiating between the working memory task conditions (i.e., zero-back, two-back). Note that the object domain activation univariate effects were not always present in the language regions. However, there might be meaningful individual differences of the activity strength. Testing whether genetic variables contribute to the individual variations of the activity strength is the goal of this line of study.

#### Cluster-level overlapping result

**Figure 4.**
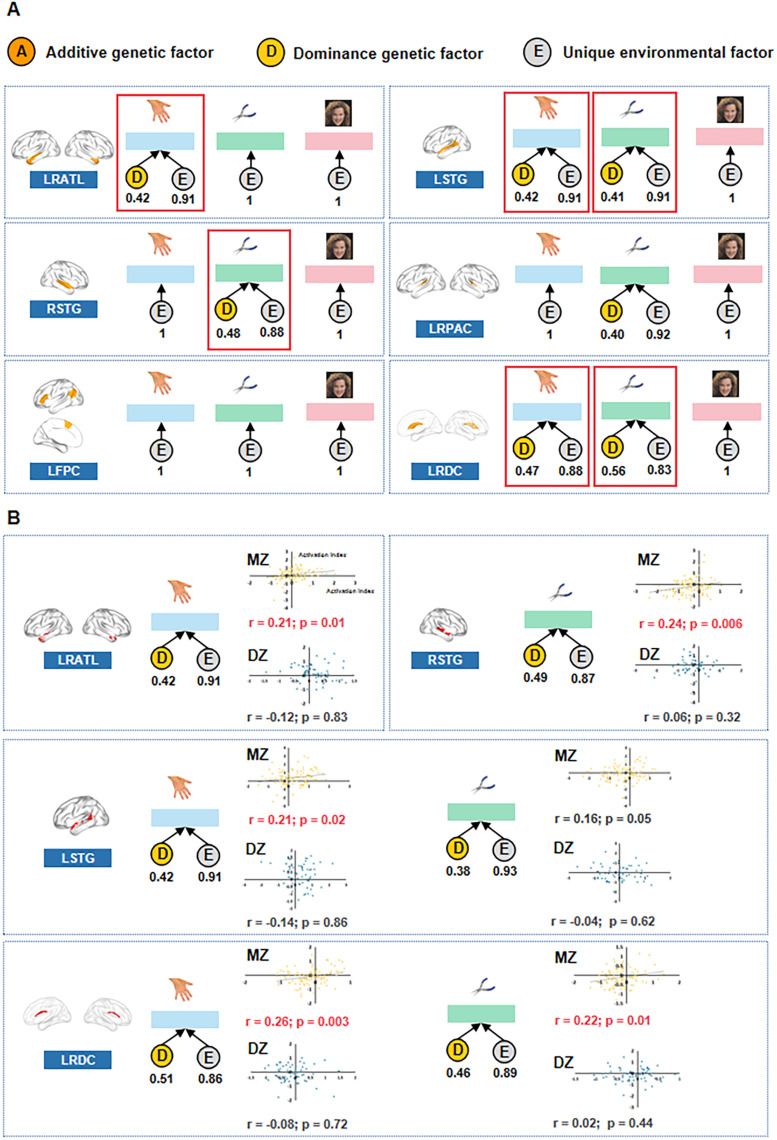
Differential genetic influence on responses to tools and body parts in different language clusters. ***A***, Genetic model results of different language clusters on responses to tools, body parts, and faces. For each language cluster of interest, the neural location is shown in the left panel. The best genetic models for face, tool, and body part processing are displayed from left to right. *A*, *D*, and *E* represent the additive genetic factor, dominance genetic factor, and unique environment factor, respectively, and their values are shown below each factor. The model with a significant genetic effect (AE model or DE model, *p* < 0.05, FDR-corrected, compared with the control model *E*) is framed with a red rectangle. See genetic model results for all the language genetic clusters after excluding specific object domain-responsive voxels in Extended Data Table 4-1. ***B***, Genetic model results at the voxel level. The cluster-level results were validated at the voxel level. For each language cluster showing a genetic effect on any object domain, genetic model analyses were conducted for the object domain of interest (with a genetic effect at the cluster level) on the “language genetic voxels.” The “language genetic voxels” were voxels with a best model that included a genetic factor (*A* or *D* factor, compared with the *E* model, at a threshold of *p* < 0.05), and they are displayed in the left panel. The best model for the object domain of interest is shown. *A*, *D*, and *E* represent the additive genetic factor, dominance genetic factor, and unique environmental factor, respectively, and their values are indicated below each factor. The scatterplots illustrate the ICC across the twin pairs in the MZ and DZ groups. Each dot represents a twin pair in each group.

We observed significant genetic effects on neural activity in the bilateral ATL in response to body parts (Fig. 4*A*, model DE, compared with the *E* model, FDR-corrected *p* = 0.044); in the left STG in response to both body parts and tools (Fig. 4*A*, body parts: model DE, compared with the *E* model, FDR-corrected *p* = 0.044; tools: model DE, compared with the *E* model, FDR-corrected *p* = 0.045); in the right STG in response to tools (Fig. 4*A*, model DE, compared with the *E* model, FDR-corrected *p* = 0.016); and in the subcortical bilateral dCa in response to both body parts and tools (Fig. 4*A*, body parts: model DE, compared with the *E* model, FDR-corrected *p* = 0.044; tools: model DE, compared with the *E* model, FDR-corrected *p* = 0.004). We did not observe a significant genetic effect on any of these specific object domains for the other language genetic clusters (bilateral primary auditory cortex and left IFG/SFG-IPL), with the best model being the *E* model or the genetic models showing no significant differences from the *E* model (Table 2; *p*s > 0.05). The genetic effect patterns persisted, even after the exclusion of voxels that showed a significant response to the respective object domain (Extended Data Table 4-1).

10.1523/ENEURO.0138-24.2024.t4-1 Table 4-1 **Genetic model results for all the language genetic clusters after excluding specific object domain responsive voxels** Note: For each language genetic cluster, the cognitive abilities with existing genetic effects (AE model or DE model, p values < 0.05, compared with the control model E, uncorrected) are marked with yellow. ΔAIC denotes the degree to which the best model is better than the control model. Download Table 4-1, DOC file.

**Table 2.** Genetic model results for all the language genetic clusters at the cluster-overlapping level

Cluster	Language			Body			Face			Tool		
	Best model	Compared with model *E*		Best model	Compared with model *E*		Best model	Compared with model *E*		Best model	Compared with model *E*	
		ΔAIC	*p*		ΔAIC	*p*		ΔAIC	*p*		ΔAIC	*p*
LRATL	DE	19.38	<0.001	DE	2.00	0.044	*E*	–	–	*E*	–	**–**
LSTG	DE	8.24	0.001	DE	1.79	0.044	*E*	–	–	DE	1.59	0.045
RSTG	DE	11.05	<0.001	*E*	–	–	*E*	–	–	DE	3.80	0.016
LRPAC	DE	5.95	0.003	*E*	–	–	*E*	–	–	DE	0.60	0.069
LFPC	AE	8.56	<0.001	*E*	–	–	*E*	–	–	*E*	–	–
LRDC	DE	6.46	0.002	DE	3.05	0.044	*E*	–	–	DE	8.39	0.004

Note: ΔAIC denotes the degree to which the best model is better than the control model. The *p* values were FDR-corrected.

Having observed overlapping genetic effects in brain activity in response to both language and tool/body part stimuli in the bilateral ATL, bilateral dCa, left STG, and right STG, we conducted the following analyses to validate their relationships.

#### Voxel-level overlapping result

We further validated the cluster-level results on the specific voxel level. Given the core interest in the language system, here for each cluster, we also defined the language genetic voxel first and examined the effects of the object domains on these voxels collectively as in an ROI approach. For the four language genetic clusters obtained above showing the overlapping results with tools and body parts, we applied genetic models to each voxel. Voxels with the best model that contained a genetic factor (*A* or *D* factor, compared with the *E* model, *p* < 0.05) were defined as “language genetic voxels.” We conducted genetic model analyses for the object domains of interest on these “language genetic voxels” for each cluster. The effects based on the cluster-level overlapping analyses were replicated in the three clusters (Fig. 4*B*; Table 3): the language genetic voxels in the ATL showed a genetic effect for responses to body parts, in the right STG for responses to tools, and in the bilateral dCa for responses to both tools and body parts. For the left STG, the language genetic voxels only showed a significant genetic effect for responses to body parts.

**Table 3.** Genetic model results for all the language genetic clusters at the voxel-overlapping level

Cluster	Language			Body			Face			Tool		
	Best model	Compared with model *E*		Best model	Compared with model *E*		Best model	Compared with model *E*		Best model	Compared with model *E*	
		ΔAIC	*p*		ΔAIC	*p*		ΔAIC	*p*		ΔAIC	*p*
LRATL	DE	34.53	<0.001	DE	2.03	0.046	*E*	–	–	*E*	–	–
LSTG	DE	17.32	<0.001	DE	1.53	0.046	*E*	–	–	DE	0.54	0.077
RSTG	DE	16.54	<0.001	*E*	–	–	*E*	–	–	DE	4.34	0.019
LRPAC	DE	13.24	<0.001	*E*	–	–	*E*	–	–	DE	0.45	0.077
LFPC	DE	14.12	<0.001	*E*	–	–	*E*	–	–	*E*	–	–
LRDC	DE	22.88	<0.001	DE	4.36	0.028	*E*	–	–	DE	2.91	0.040

Note: ΔAIC denotes the degree to which the best model is better than the control model. The *p* values were FDR-corrected.

#### CP model result

**Figure 5.**
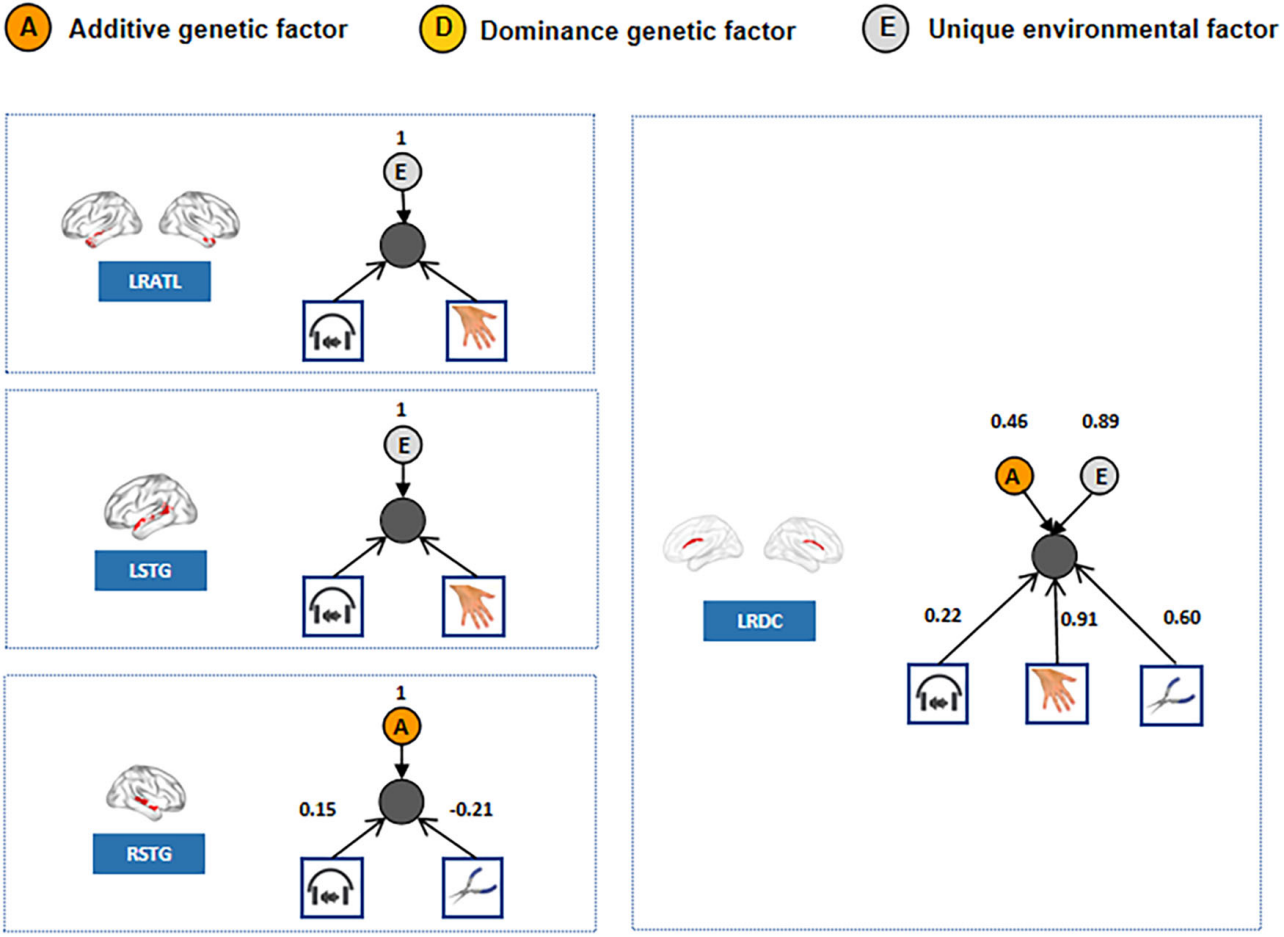
CP model results. For each language cluster of interest, the neural location is shown in the left panel. The activation indexes of the language (indicated by the headphone icon) and the object domain with a genetic effect are fitted into the CP model to determine whether the common factor has a genetic influence. The resulting model is shown in the right panel. *A*, *D*, and *E* denote the additive genetic factor, dominance genetic factor, and unique environment factor, respectively. The factor value is shown above it. The value between cognitive ability (i.e., language and object domains) and common factors represent the loading value of each cognitive capability on the common factor.

To directly assess the shared genetic component between language and specific object domains, we utilized the CP model, which allowed us to test whether genes and the environment worked through one or multiple common latent factors (Bates et al., 2019). With an optimal common factor number of one, we fitted the existing genetic influence on language and object domains to the CP model to determine if the common factor was influenced by a genetic factor. We focused on the clusters and object domains with consistent effects at both the cluster level and the voxel level. Results are shown in Figure 5. For the bilateral dCa, the common factor generated from language, body parts, and tools was influenced by genetic factor *A*. For the right STG, the common factor generated from language and tools was influenced by genetic factor *A*, with a negative loading indicating that the directions of language and tool activation is oppositely associated. For the bilateral ATL and the left STG, no significant genetic factor was found to influence the common factor between language and body parts.

That is, the overlapping effect indeed arose from shared genetic components between responses to language and both body parts and tool processing in the bilateral dCa and between language and tool processing in the right STG. The overlapping effects between language and body parts in the ATL and in the left STG may not arise from the shared genetic component.

#### Validation using anatomically defined ROIs

To examine the robustness of the effects in the two clusters obtained above (bilateral dCa and right STG) across different parcellations, we performed a validation analysis using the HCPex template (Huang et al., 2022). The HCPex is a modified and extended version of the original atlas (HCP-MMP v1.0; Glasser et al., 2016) in which 66 subcortical areas (33 in each hemisphere) covering the basal ganglia have been added. The bilateral dCa cluster corresponded to the combination of Caudate_L and Caudate_R regions. The right STG cluster corresponded to the combination of Area_STGa_R, Area_STSd_anterior_R, Area_STSd_posterior_R, Area_STSv_anterior_R, and Area_STSv_posterior_R. We reestimated the genetic effects of language and object domain processing using the HCPex template and obtained a consistent result pattern (bilateral basal ganglia: language, model DE vs model *E*, *p* = 0.040, tools, model DE vs model *E*, *p* < 0.001, body parts, model DE vs model *E*, *p* = 0.0067; right STG: language, model DE vs model *E*, *p* < 0.001, tools, model AE vs model *E*, *p* = 0.001).

#### Validation by regressing out handedness

To enhance the reliability of the data, we did not distinguish between different handedness groups in the main analysis (Schmitt et al., 2014; Le Guen et al., 2018). To examine whether the results were affected by handedness, we controlled for handedness information (provided by the HCP) when estimating the genetic models. A similar pattern of results was obtained after controlling for the handedness information (bilateral basal ganglia: language, model DE vs model *E*, *p* = 0.004, tools, model AE vs model *E*, *p* = 0.003, body parts, model DE vs model *E*, *p* = 0.026; right STG: language, model DE vs model *E*, *p* < 0.001, tools, model DE vs model *E*, *p* = 0.011).

## Discussion

To understand the genetic basis of the neural activity of the complex language brain regions, we investigated the patterns in which they shared genetic effects among each other and with a set of information domains that have been proposed to relate to language in evolution: tools, faces (social), and body parts (social and gesturing). We used the twin datasets released by the HCP that had fMRI data obtained from twins (MZ and DZ) when performing language task and working memory task on various object domains. We identified the neural activity responding to language in a large set of cortical regions (mainly located in the frontal and temporal cortices) and subcortical regions (basal ganglia) that exhibited genetic effects, with both common and different genetic expression profiles. Among them, the bilateral basal ganglia (mainly dCa) showed a common genetic basis for language, tool, and body part processing, and the right STG showed a common genetic basis for language and tool processing across multiple types of analyses.

The finding that neural activity to language is genetically influenced is certainly expected. Language behaviors have long been linked with genetics (Doust et al., 2022; Eising et al., 2022; Mekki et al., 2022; Carrión-Castillo et al., 2023). Previous reports looking at brain responses with HCP data have shown that the STS and IFG were influenced by genetics (Le Guen et al., 2018), convergent with our cortical findings here. Importantly, in addition to the cortical language regions, we further observed that subcortical structures, specifically the dCa of the basal ganglia, also showed genetically driven language responses, aligning with the recent recognition of its role in language processing from neuroimaging and neuropsychological studies (Kotz et al., 2009; Thibault et al., 2021; Murphy et al., 2022). More broadly, we revealed commonality and important heterogeneity among the genetic effects in the largely distributed language network. For commonality, they responded to language and displayed genetic expression profiles systematically different from the rest of the brain. For heterogeneity, these clusters also showed genetic expressions that differ from each other and different association profiles with the genetic effects of related object domains.

Critically, one key finding here was the discovery of a shared genetic basis for language with tools and body parts in the dCa and for language with tools in the right STG. While language processing entails an array of cognitive functions (Hauser et al., 2002; Ben Shalom and Poeppel, 2008), the current interest in genetic effects motivated us to focus on those processes that have been proposed to be associated with language evolution—tool use, gesturing, and social processes (Fitch et al., 2010; Vaesen, 2012; Rajimehr et al., 2022). The process of evolution underlying the emergence of differences between species was accompanied by natural selection acting on the available genetic diversity (Roberts, 2022). These findings about different genetic effect patterns for related processes across the various language-sensitive brain regions, with distinct genetic expression profiles, suggest different genetic bases among these regions with potential implications for language evolution.

For the basal ganglia (dCa), not only did its responses to language, tools, and body parts all show overlapping genetic effects at both the cluster and voxel levels, but a common factor explained the variance influenced by genetics across these processes. This finding resonates with the recent discovery of shared neural representation for language and tool use in this region (Thibault et al., 2021), providing novel genetic evidence and extending to body part processing. What do these processes have in common to be the potential shared genetic functionality here? For the association between language and tools, Brozzoli et al. (2019) found that, after ruling out the influence of manual or linguistic motor skills, individual motor proficiency during tool use predicted sentence production performance. Thibault et al. (2021) further found that complex syntax in language (object relative clauses vs coordinated clauses and subjective clauses) had overlapping activity with tool-use planning in the bilateral internal globus pallidus and left caudate, with the latter also showing a correlation pattern between free-hand planning and sentence comprehension. The finding that the genetic component shared by tool use and language was also shared with body part processing (and not face processing) was further illuminating. Whatever underlying the genetically supported functionality is served by dCa, it plays a role in body parts too. It is not likely to be social or conspecific functions, as the effect did not generalize to face processing; it is not likely to be simply motor effects, as the motor function is also involved in the control conditions across these studies; and it is not likely to be related to task-general effects such as working memory, attention, or visual processing, as we used the “place” domain as the control baseline when considering tools and body parts in the same working memory task and that “face” domain did not show similar effects. A common hypothesis about language–tool associations has been related to hierarchical information processing. For language, in sentence parsing/construction, especially in complex structures such as sentences with object clauses, processing elements entails more complex dependency relations. For gesturing, it entails complex dependency between multiple motor actions. For tool use, incorporating the external object also adds additional dependency structures—the relationship between the body and the tool and the tool and other target objects needed to be orchestrated in the context of a goal-driven process accordingly. The body part effects we observed here could arise either from gesturing implications or from embodying tool use (simulations) with body parts or both.

The right STG showed a shared genetic component between language and tool processing yet with a negative loading indicating that the directions of language and tool activation are oppositely associated. For tool relevance, this region has been implicated in visual (motion) and sound of tools and audiovisual integration (Beauchamp, Argall, et al., 2004; Beauchamp, Lee, et al., 2004; Lewis et al., 2005; Stevenson and James, 2009; Han et al., 2013). For language relevance, this region has been implicated in syntactic processing, language comprehension, and audiovisual integration in speech recognition (Friederici et al., 2009; Stevenson and James, 2009; Zaccarella et al., 2015). However, it is not obvious what kind of process may affect language and tool in opposite ways.

Genetic influence affected responses to language, tools, and body parts in the left STG without a significant CP effect and affected responses to language and body parts in the bilateral ATL without a significant CP effect. These result patterns are taken to suggest overlapping genetic effects across language and these object domains in these two regions but are not necessarily driven by the same genetic mechanisms. For the bilateral ATL, suggested functions based on imaging results included semantic representation, combination, and social processes (Pylkkänen, 2019; Hung, 2020; X. Wang et al., 2020; Fu et al., 2023). The genetic effect of body part responses does not evidently arise from a shared genetic component with language (or face and tools) and thus does not lend support for a genetic language semantic effect arising from gesturing here. For the left STG, the results suggested that processing of body-related functions supporting tools and body parts here may not share genetic mechanisms with language either.

Two clusters—the bilateral primary auditory cortex and the left IFG/SFG-IPL—did not show genetic effects in responses to tools, body parts, or face processes. While it is tempting to conclude that these regions are genetically “language-specific,” it is important to emphasize that we could not rule out false-negative possibilities. The bilateral primary auditory cortex is not a traditional language region, and the results here may simply reflect the genetic effects of general auditory responses. The object domain effects were derived from a picture working memory task that may not engage strongly enough in the hypothesized dimensions (tool use, gesturing, social interaction). While viewing tools and bodies elicit large-scale brain networks beyond the visual ventral cortex that overlap with related actions (tool use and body action in the frontal and parietal cortices, respectively; Lewis, 2006; Bracci and Peelen, 2013; Van Den Heiligenberg et al., 2018), it is always possible that the specific task engagement is not optimal to reveal such effects. Along the same line, we did not see any language genetic clusters showing overlap with genetic effects of face processing, the interpretation of such negative results require caution. Previous studies have shown that the genetic effects of brain activity to faces seems to be weaker compared with other object domains or general capability (Abbasi et al., 2020; Quinones Sanchez et al., 2021), indicating that the current brain activity measure (univariate contrast) may not be optimal to reflect face processing neural mechanisms. We did not explore the regions beyond the language network for such association either due to the current focus on the language network. More generally, we do not wish to use negative results to claim against potential shared origins between language and other cognitive domains.

There are also regions that showed a preferential response to language but exhibited a significant effect of common environmental variables, including specific clusters in the left MFG, IFG, OrG, PrG, and STG (Fig. 2*A*). Note that these clusters were not reported in previous studies looking at genetic effects of language activities because they included nontwin siblings and thus did not investigate environmental effects (Le Guen et al., 2018). These environmentally influenced language regions we discovered were in close proximity to the genetically influenced language regions, suggesting possible tight interactions with each other. The nature of the environmental effects (e.g., socioeconomic status and language exposure) warrants further investigation.

To conclude, while a range of brain cortical and subcortical regions' responses to language are genetically affected, they show different genetic effect profiles in terms of a shared genetic basis with processing specific object domains. The brain responses in a basal ganglia region (dCa) to language, tools, and body parts have common genetic effects and brain responses in the right STG to language and tools. These results uncover the heterogeneous genetic patterns of language neural processes, shedding light on the evolution of language and its shared origins with tools and bodily functions.

## Synthesis

Reviewing Editor: James Howard, Brandeis University Decisions are customarily a result of the Reviewing Editor and the peer reviewers coming together and discussing their recommendations until a consensus is reached. When revisions are invited, a fact-based synthesis statement explaining their decision and outlining what is needed to prepare a revision will be listed below. The following reviewer(s) agreed to reveal their identity: Reza Rajimehr. Note: If this manuscript was transferred from JNeurosci and a decision was made to accept the manuscript without peer review, a brief statement to this effect will instead be what is listed below.

In one major comment, a reviewer asked the authors to provide group-average activation maps for bodies, faces, and tools. In the figure shown in the response letter, it is not clear what the maps are (color scale bars are not included). Assuming that the red/yellow regions show significant activations, it is clear that part of the language mask overlaps with activations for bodies and faces. Thus, some of the reported genetic effects for bodies may simply be due to the presence of body-responsive voxels in the language mask. If the authors exclude those voxels in the analysis, the genetic effects may disappear. We ask the authors to carefully evaluate this, and if it is true, clearly discuss about it in the manuscript.
