# Supplementary material for: Processing Language Partly Shares Neural Genetic Basis with Processing Tools and Body Parts
Source: eNeuro. 2024 Aug 1;11(8):ENEURO.0138-24.2024. doi: 10.1523/ENEURO.0138-24.2024 (PMC11298957; doi:10.1523/ENEURO.0138-24.2024)
Supplement: Figure 2-1 — Language genetic influence result without a language mask A. Multiple types of whole-brain regions. After genetic modeling without restricting the language activation map, multiple types of regions were identified. B. The clustering result of the genetic regions without a language activation mask. Download figure 2-1, DOCX file. [file eneuro-11-ENEURO.0138-24.2024-s001.docx]

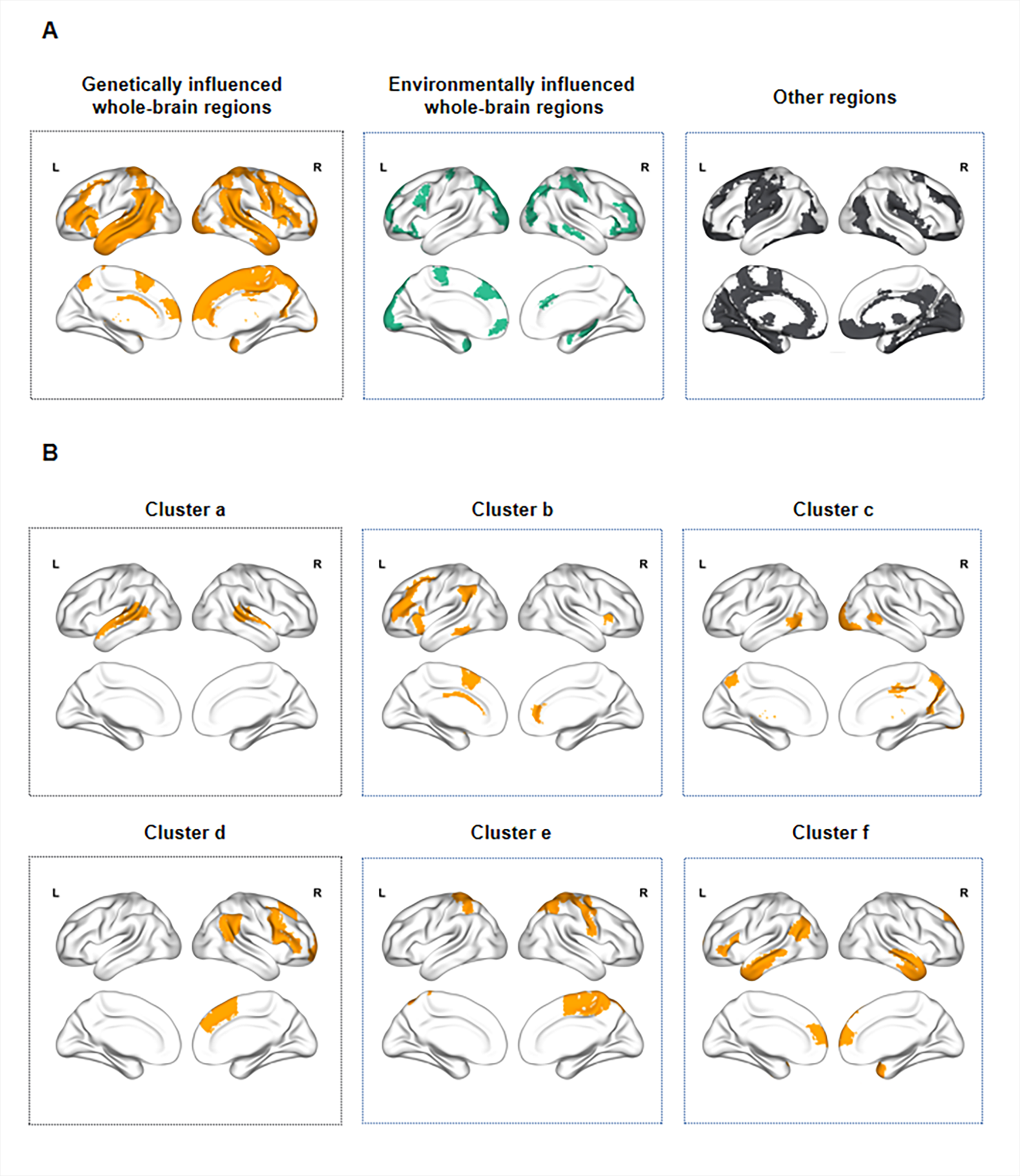


**Figure 2-1| Language genetic influence result without a language mask**

1. **Multiple types of whole-brain regions.** After genetic modeling without restricting the language activation map, multiple types of regions were identified.

**The clustering result of the genetic regions without a language activation mask.**
