# Supplementary material for: Processing Language Partly Shares Neural Genetic Basis with Processing Tools and Body Parts
Source: eNeuro. 2024 Aug 1;11(8):ENEURO.0138-24.2024. doi: 10.1523/ENEURO.0138-24.2024 (PMC11298957; doi:10.1523/ENEURO.0138-24.2024)
Supplement: Table 3-1 — Functional labels based on Neurosynth for genes contributing to commonality of language clusters Note: For each gene, the functional terms from Neurosynth represent the terms with the most similar meta-analysis whole-brain activation map to the gene’s whole-brain map. The correlation values indicate the correlation of the gene’s whole-brain expression with the term’s meta-analysis result. Download Table 3-1, DOC file. [file eneuro-11-ENEURO.0138-24.2024-s002.doc]

**Table 3-1. Functional labels based on Neurosynth for genes contributing to commonality of language clusters**

| Gene name | Function 1 | R | Function 2 | R |
| --- | --- | --- | --- | --- |
| TCAP | Dementia | 0.093 | Social | 0.092 |
| KCNE4 | Reward | 0.167 | Anticipation | 0.151 |
| LGALS2 | [Motor imagery](https://www.neurosynth.org/analyses/terms/motor imagery) | 0.142 | [Sensorimotor](https://www.neurosynth.org/analyses/terms/sensorimotor) | 0.137 |
| FREM3 | Semantic | 0.153 | Language | 0.141 |
| MEDAG | - | - | - | - |

Note: For each gene, the functional terms from Neurosynth represent the terms with the most similar meta-analysis whole-brain activation map to the gene’s whole-brain map. The correlation values indicate the correlation of the gene’s whole-brain expression with the term’s meta-analysis result.
