# Supplementary material for: Processing Language Partly Shares Neural Genetic Basis with Processing Tools and Body Parts
Source: eNeuro. 2024 Aug 1;11(8):ENEURO.0138-24.2024. doi: 10.1523/ENEURO.0138-24.2024 (PMC11298957; doi:10.1523/ENEURO.0138-24.2024)
Supplement: Table 3-2 — Functional labels based on Neurosynth for genes contributing to differences of different language clusters Note: For each gene, the functional terms from Neurosynth represent the terms with the most similar meta-analysis whole-brain activation map to the gene’s whole-brain map. The correlation values indicate the correlation of the gene’s whole-brain expression with the term’s meta-analysis result. Download Table 3-2, DOC file. [file eneuro-11-ENEURO.0138-24.2024-s003.doc]

**Table 3-2. Functional labels based on Neurosynth for genes contributing to differences of different language clusters**

| Cluster  name | Gene name | Function 1 | R | Function 2 | R | Cluster  name | Gene name | Function 1 | R | Function 2 | R |
| --- | --- | --- | --- | --- | --- | --- | --- | --- | --- | --- | --- |
| Cluster 1 | Bilateral anterior temporal lobe | | | | | Cluster 2 | Left superior temporal gyrus | | | | |
|  | KCNG1 | Emotional | 0.165 | Neutral | 0.145 |  | SLC25A12 | Visual | 0.206 | [Somatosensory](https://www.neurosynth.org/analyses/terms/somatosensory) | 0.133 |
|  | PRKCG | [Encoding](https://www.neurosynth.org/analyses/terms/encoding) | 0.21 | [Episodic](https://www.neurosynth.org/analyses/terms/episodic) | 0.204 |  | STAU2 | Visual | 0.138 | Tasks | 0.115 |
|  | PTGER3 | Neutral | 0.339 | Fearful | 0.327 |  | RIMKLA | Hand | 0.169 | Motor | 0.167 |
|  | TP53I11 | Dementia | 0.119 | Social | 0.103 |  | MARF1 | - | - | - | - |
|  | HTR1A | Semantic | 0.14 | Dementia | 0.135 |  | GOLGA7B | Retrieval | 0.103 | Tasks | 0.101 |
| Cluster 3 | Right superior temporal gyrus | | | | | Cluster 4 | Bilateral primary auditory cortex | | | | |
|  | CENPF | Action | 0.118 | Dementia | 0.106 |  | BHLHE22 | Encoding | 0.19 | Episodic memory | 0.143 |
|  | RASAL1 | Semantic | 0.124 | Retrieval | 0.113 |  | VAV3 | Motor | 0.166 | Visual | 0.14 |
|  | GYPE | Semantic | 0.165 | Language | 0.144 |  | GLCCI1 | Visual | 0.163 | Hand | 0.084 |
|  | SCN3B | Retrieval | 0.161 | Episodic | 0.144 |  | IGFBP2 | Language | 0.144 | Visual | 0.139 |
|  | ATOH7 | Semantic | 0.115 | Memories | 0.114 |  | WNT3 | [Nociceptive](https://www.neurosynth.org/analyses/terms/nociceptive) | 0.109 | Pain | 0.108 |
| Cluster 5 | Left frontal-parietal cortex | | | | | Cluster 6 | Bilateral dorsal caudate | | | | |
|  | LCP2 | Motor | 0.158 | Hand | 0.149 |  | KLHL13 | Reward | 0.275 | Incentive | 0.238 |
|  | CTXN3 | Task | 0.157 | Working memory | 0.133 |  | NEXN | Motor | 0.111 | Finger tapping | 0.108 |
|  | GAL | Motor | 0.145 | Pain | 0.134 |  | HEYL | Reward | 0.24 | Anticipation | 0.207 |
|  | HAPLN4 | Motor | 0.182 | Hand | 0.18 |  | COCH | Incentive | 0.343 | Reward | 0.337 |
|  | GPR20 | Motor | 0.234 | Movements | 0.223 |  | PCP4 | Incentive | 0.211 | Motor | 0.206 |

Note: For each gene, the functional terms from Neurosynth represent the terms with the most similar meta-analysis whole-brain activation map to the gene’s whole-brain map. The correlation values indicate the correlation of the gene’s whole-brain expression with the term’s meta-analysis result.
