# Supplementary material for: Processing Language Partly Shares Neural Genetic Basis with Processing Tools and Body Parts
Source: eNeuro. 2024 Aug 1;11(8):ENEURO.0138-24.2024. doi: 10.1523/ENEURO.0138-24.2024 (PMC11298957; doi:10.1523/ENEURO.0138-24.2024)
Supplement: Table 4-1 — Genetic model results for all the language genetic clusters after excluding specific object domain responsive voxels Note: For each language genetic cluster, the cognitive abilities with existing genetic effects (AE model or DE model, p values < 0.05, compared with the control model E, uncorrected) are marked with yellow. ΔAIC denotes the degree to which the best model is better than the control model. Download Table 4-1, DOC file. [file eneuro-11-ENEURO.0138-24.2024-s004.doc]

**Extended table 4-1. Genetic model results for all the language genetic clusters after excluding specific object domain responsive voxels**

| Cluster | Body | | | Face | | | Tool | | |
| --- | --- | --- | --- | --- | --- | --- | --- | --- | --- |
|  | Best model | Compared with model E | | Best model | Compared with model E | | Best model | Compared with model E | |
|  |  | ∆AIC | P |  | ∆AIC | P |  | ∆AIC | P |
| LRATL | DE | 2.15 | 0.017 | E | - | - | E | - | **-** |
| LSTG | DE | 0.83 | 0.039 | E | - | - | DE | 1.56 | 0.023 |
| RSTG | E | - | - | E | - | - | DE | 3.79 | 0.005 |
| LRPAC | E | - | - | E | - | - | DE | 0.53 | 0.047 |
| LFPC | E | - | - | E | - | - | E | - | - |
| LRDC | DE | 3.55 | 0.009 | E | - | - | DE | 8.39 | 0.0006 |

Note: ∆AIC denotes the degree to which the best model is better than the control model.
